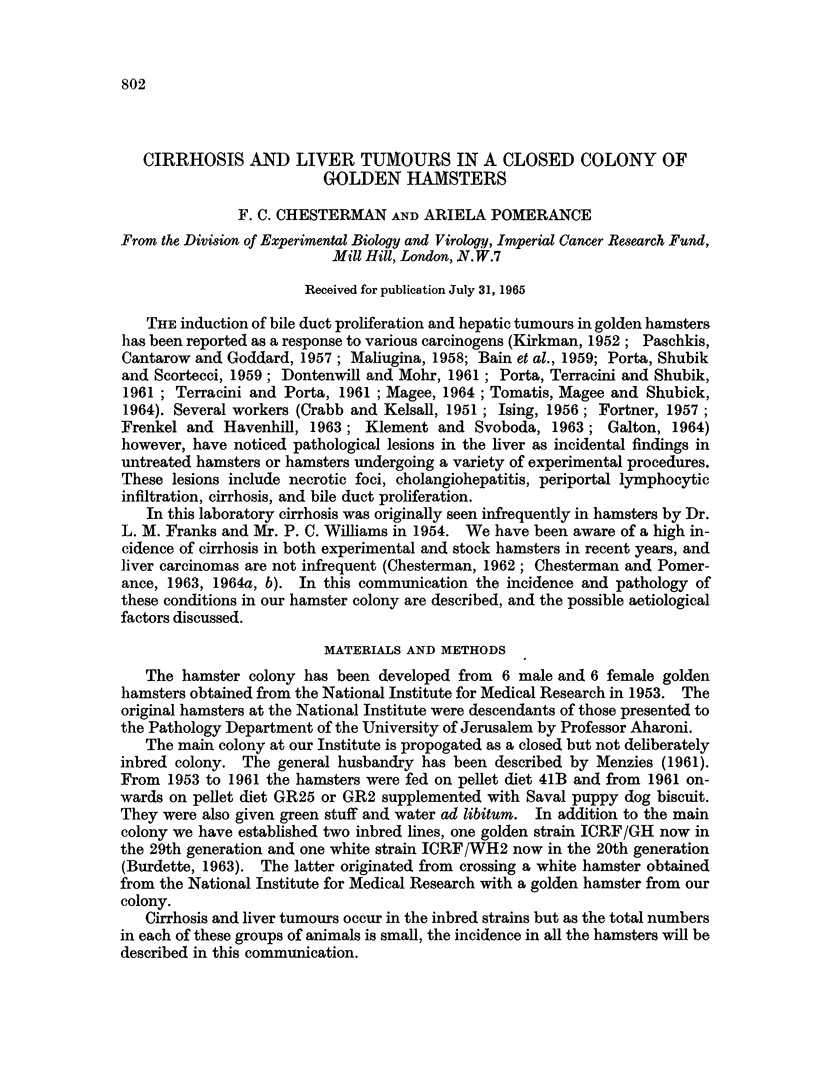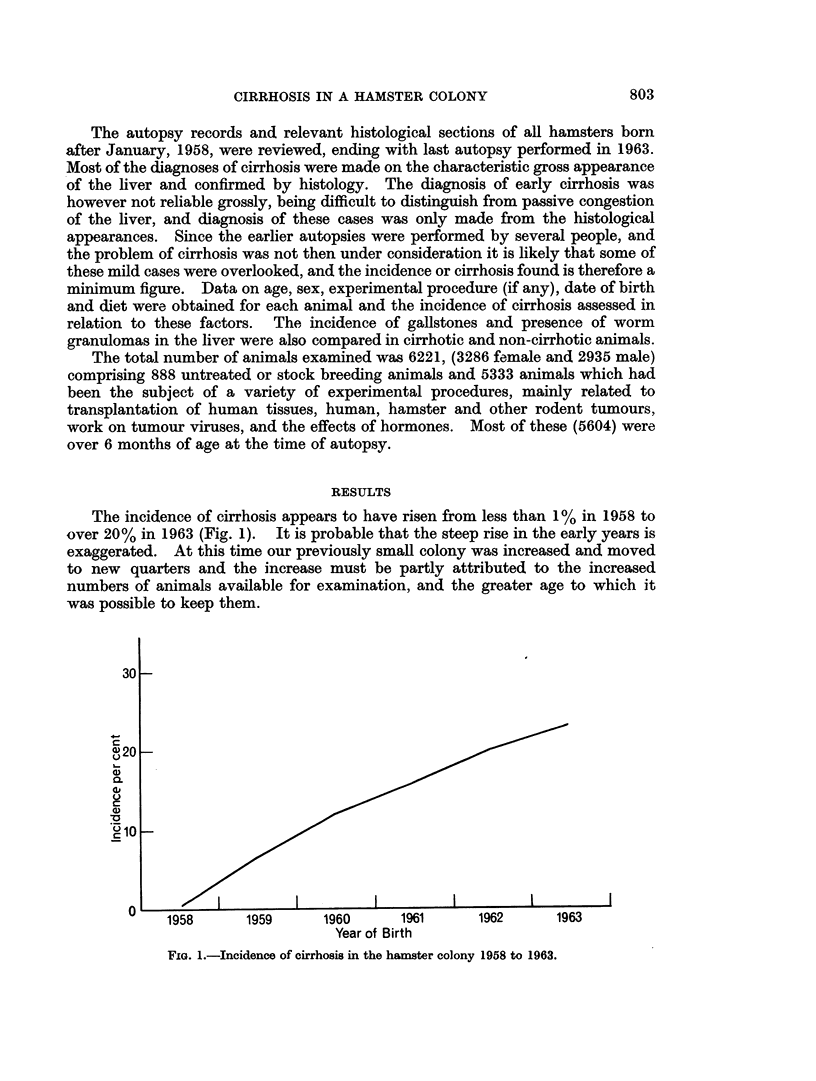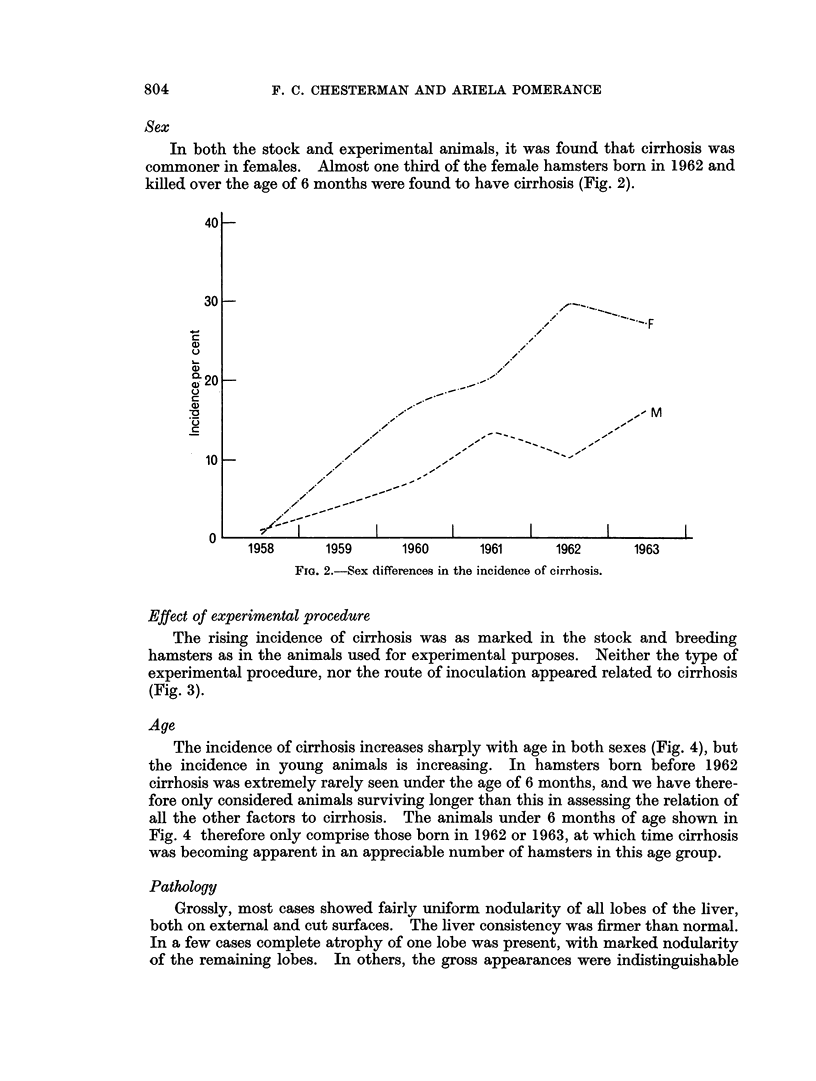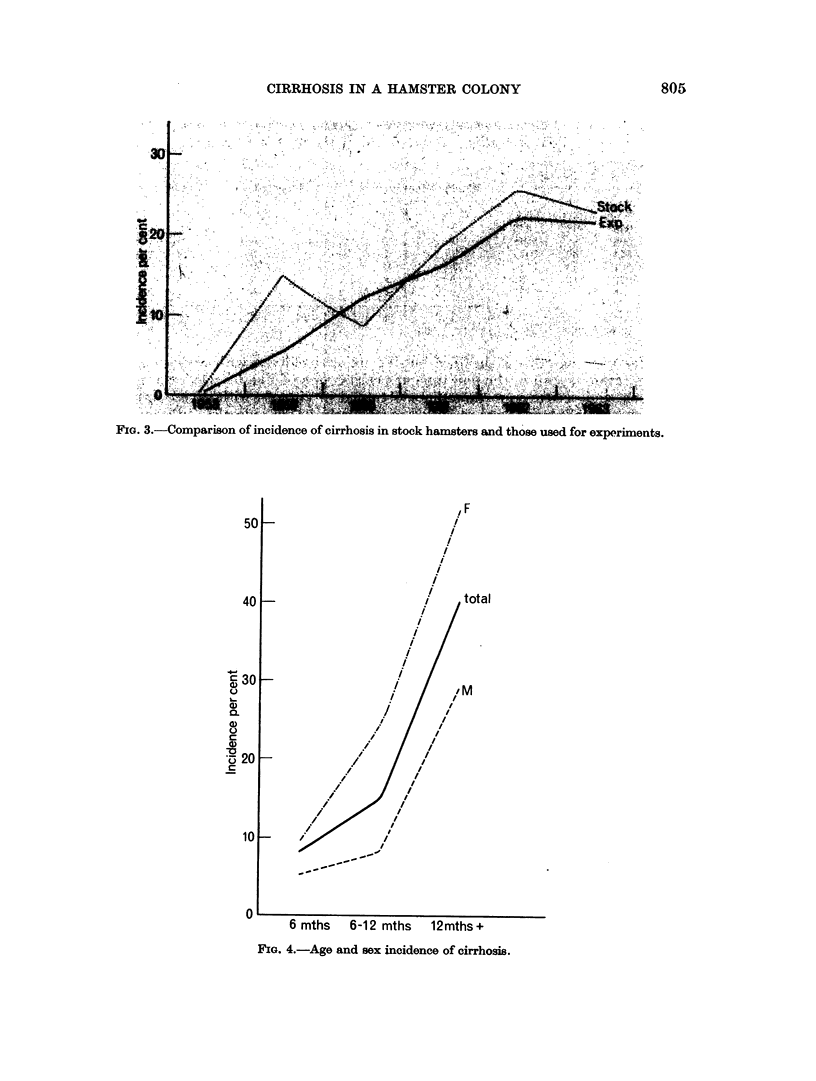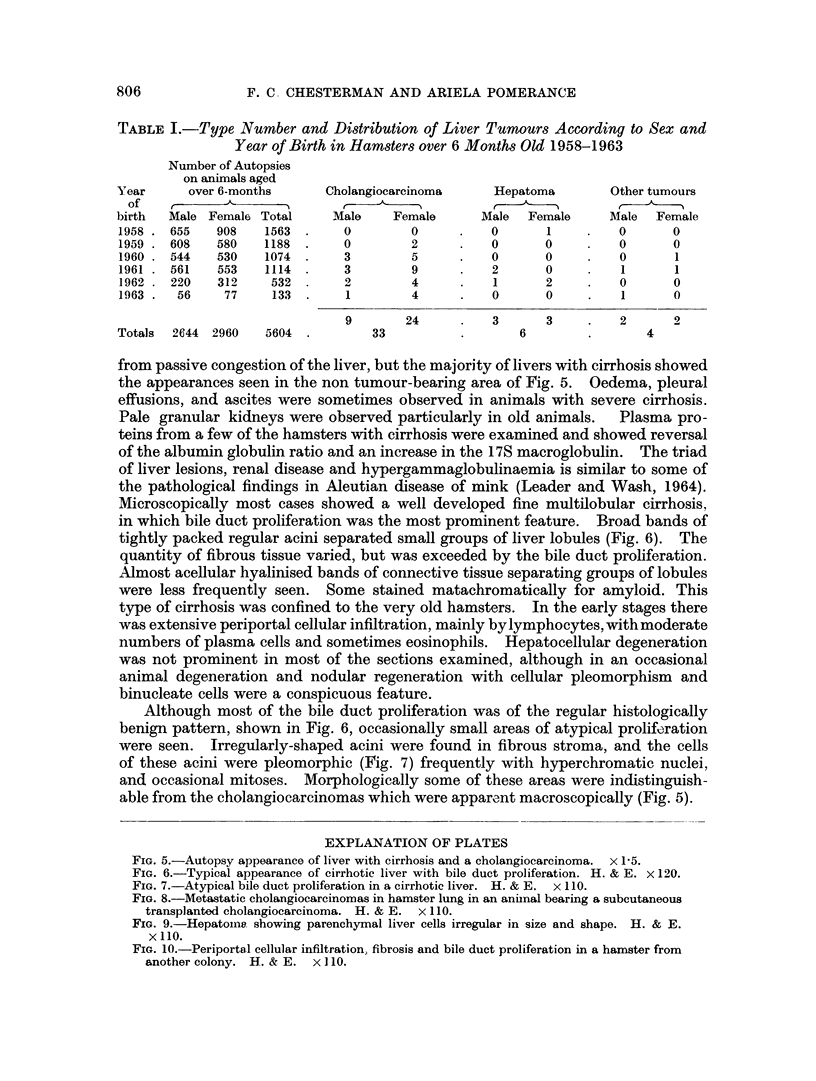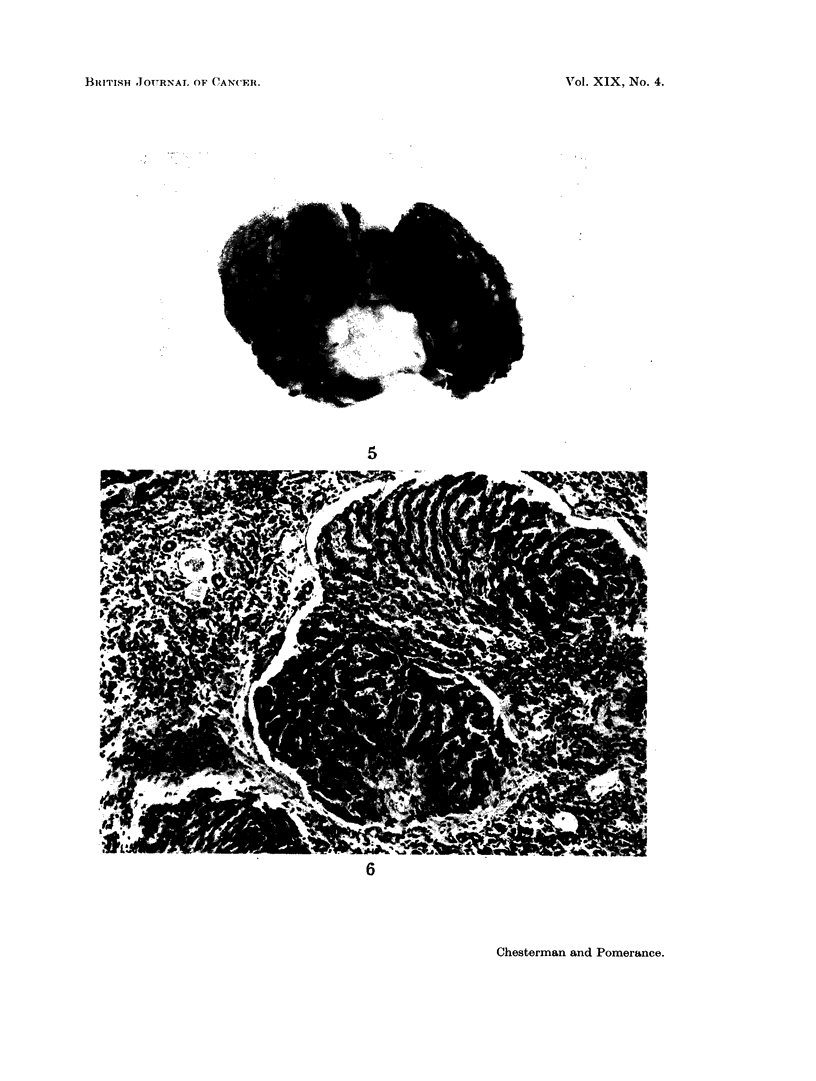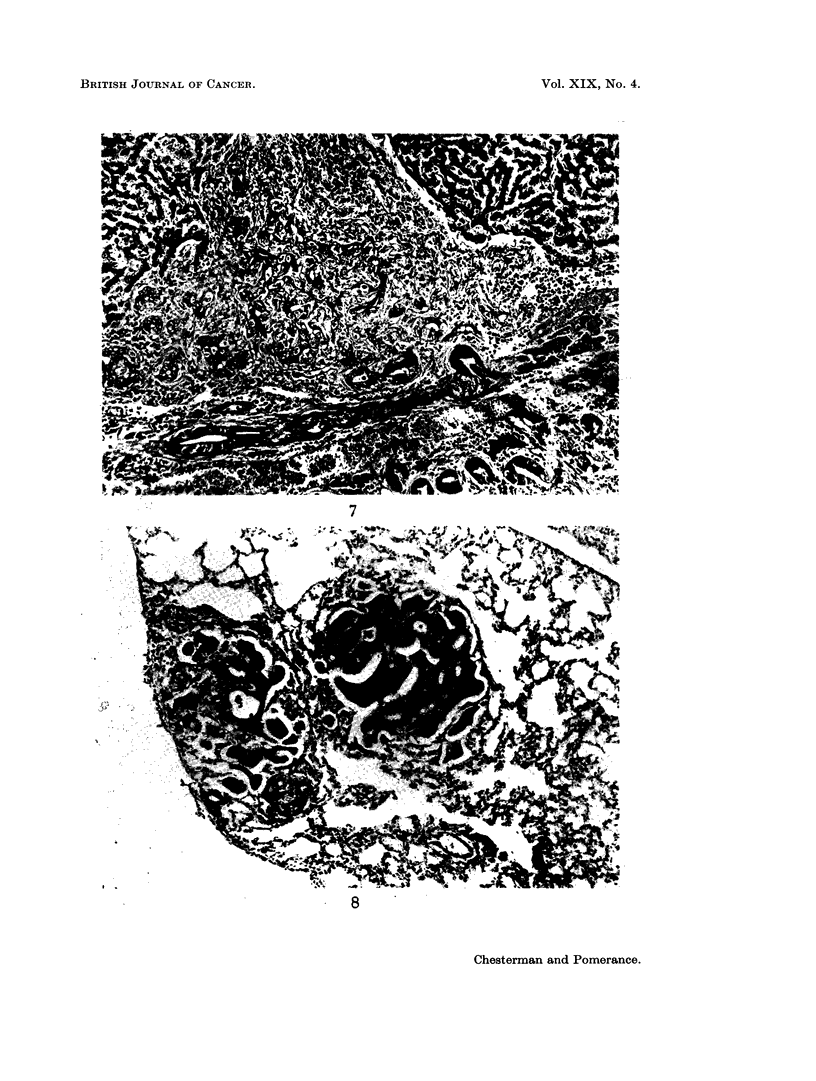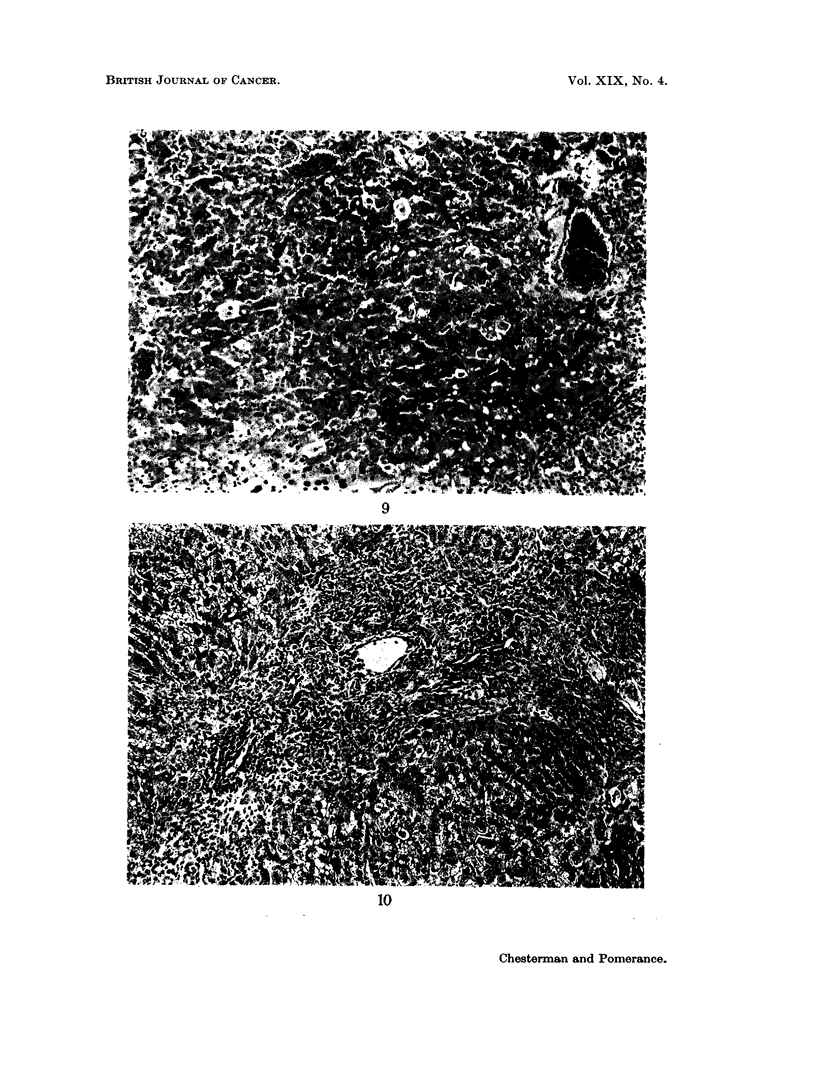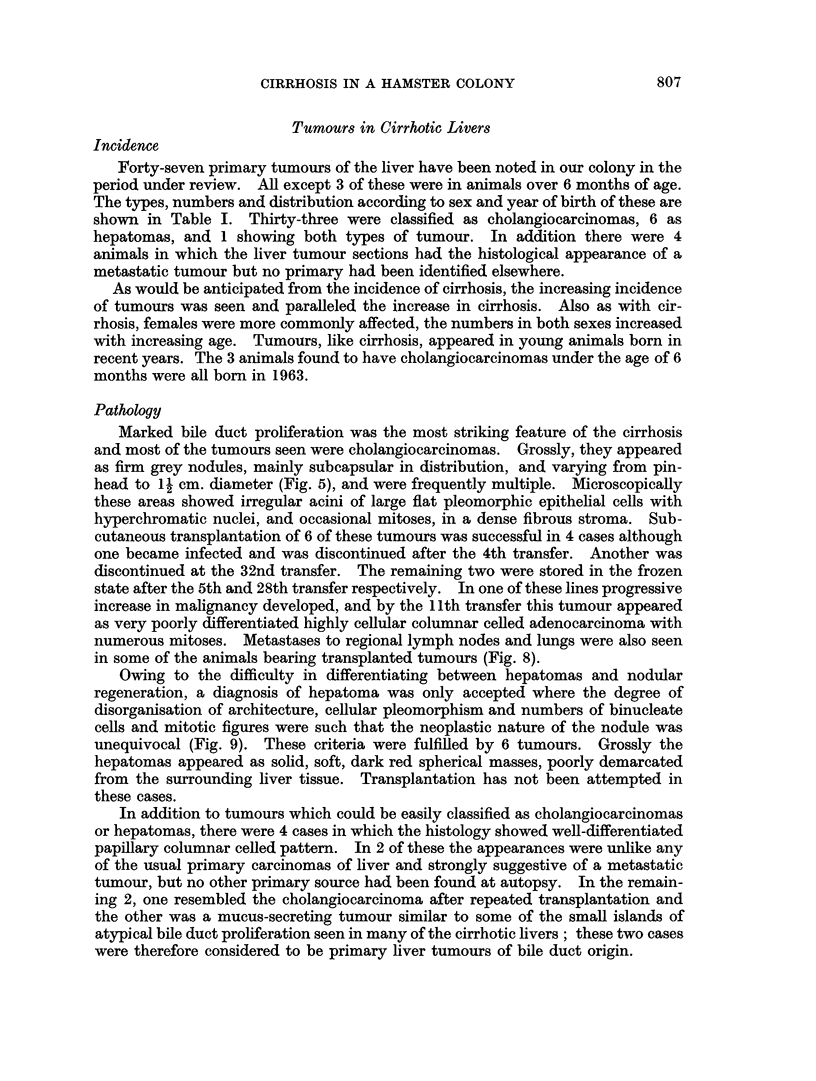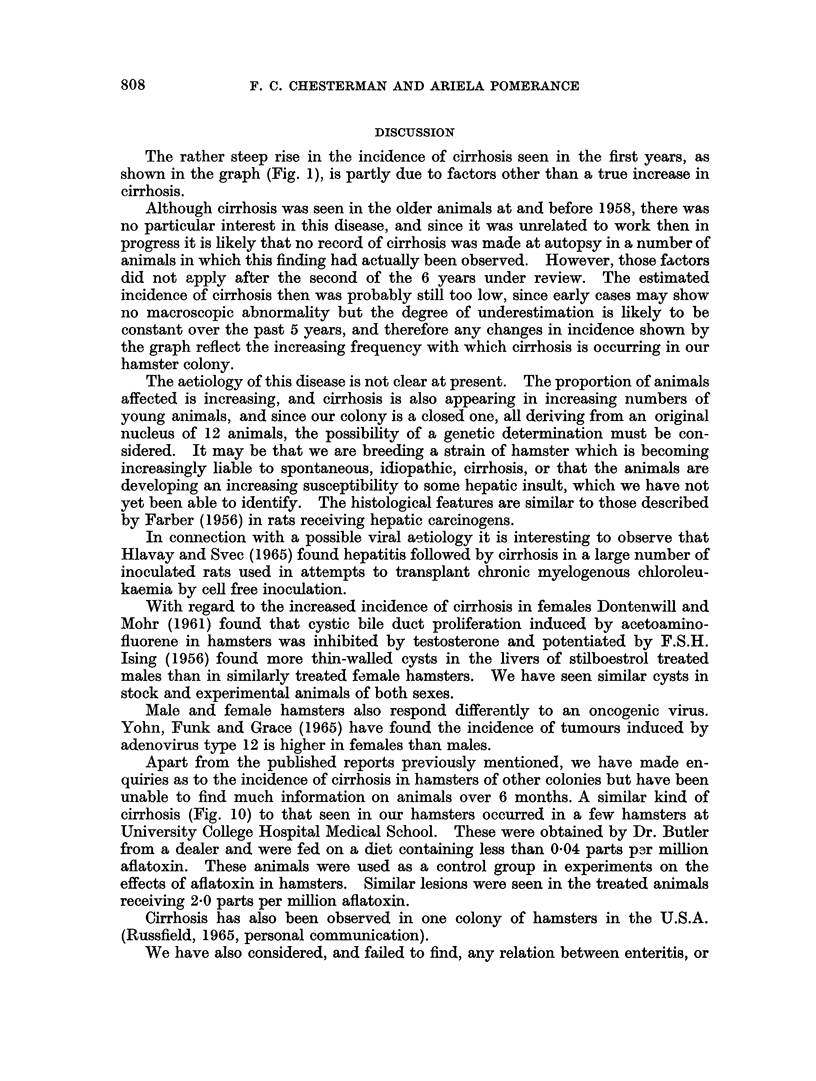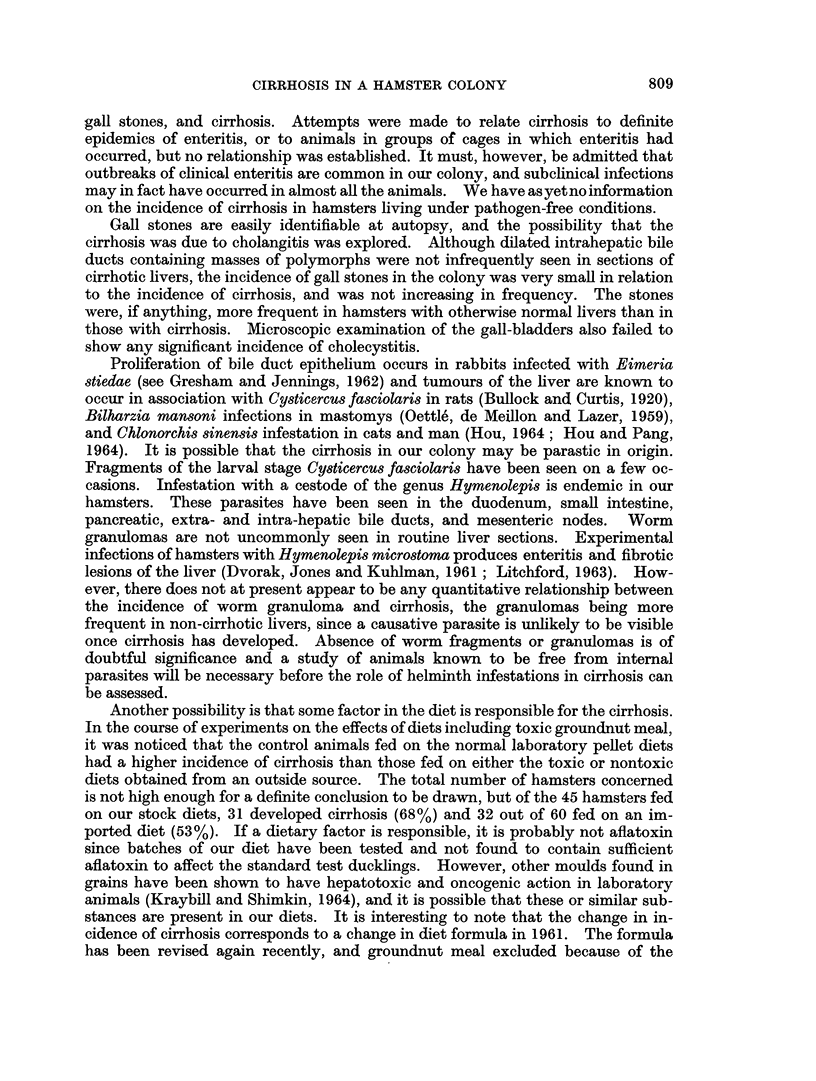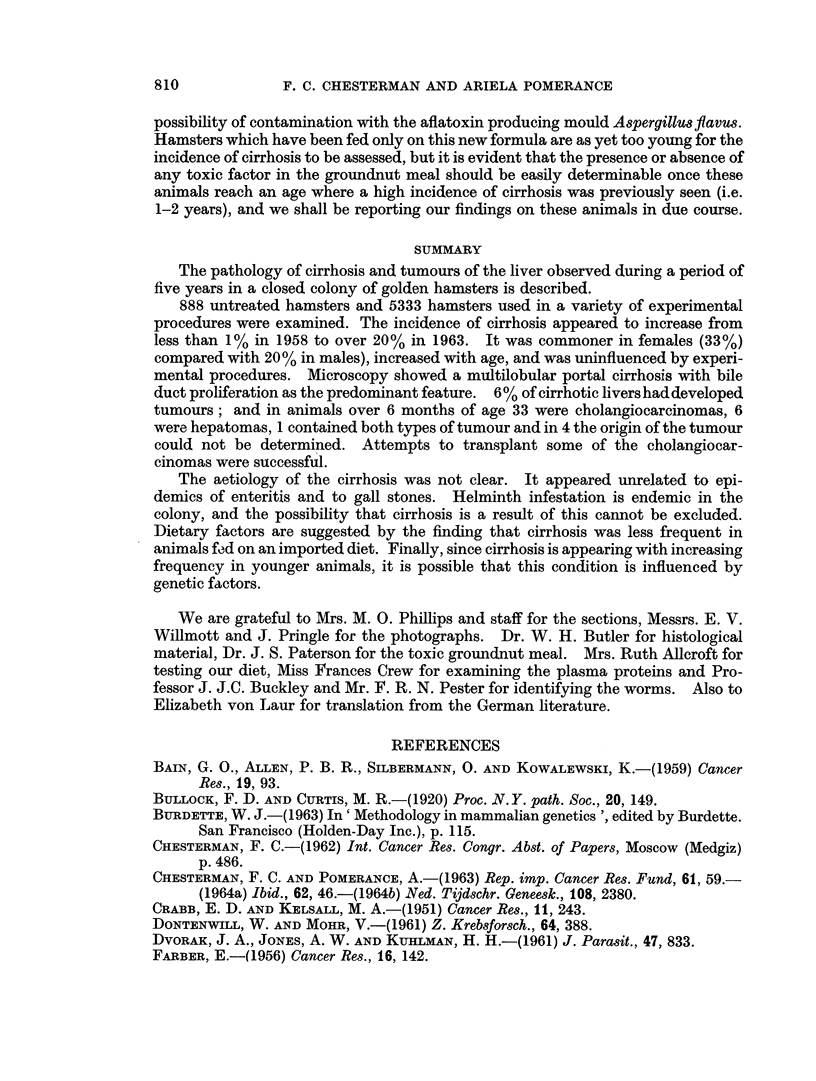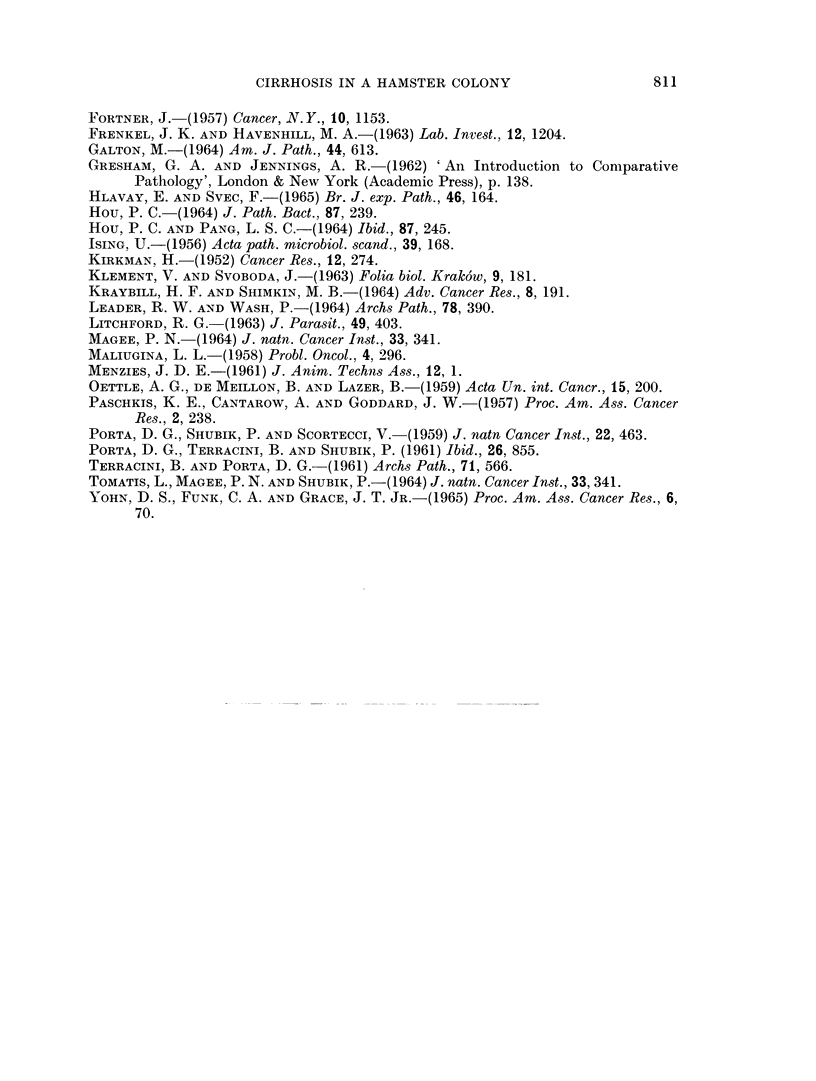# Cirrhosis and liver tumours in a closed colony of golden hamsters.

**DOI:** 10.1038/bjc.1965.92

**Published:** 1965-12

**Authors:** F. C. Chesterman, A. Pomerance

## Abstract

**Images:**


### CIRRHOSIS AND LIVER TUMOURS IN A CLOSED COLONY OF GOLDEN HAMSTERS

F. C. CHESTERMAN AND ARIELA POMERANCE

*From the Division of Experimental Biology and Virology, Imperial Cancer Research Fund, Mill Hill, London, N.W.7*

Received for publication July 31, 1965

THE induction of bile duct proliferation and hepatic tumours in golden hamsters has been reported as a response to various carcinogens (Kirkman, 1952 ; Paschkis, Cantarow and Goddard, 1957 ; Maliugina, 1958; Bain *et al.*, 1959; Porta, Shubik and Scortecci, 1959 ; Dontenwill and Mohr, 1961 ; Porta, Terracini and Shubik, 1961 ; Terracini and Porta, 1961 ; Magee, 1964 ; Tomatis, Magee and Shubick, 1964). Several workers (Crabb and Kelsall, 1951 ; Ising, 1956 ; Fortner, 1957 ; Frenkel and Havenhill, 1963 ; Klement and Svoboda, 1963 ; Galton, 1964) however, have noticed pathological lesions in the liver as incidental findings in untreated hamsters or hamsters undergoing a variety of experimental procedures. These lesions include necrotic foci, cholangiohepatitis, periportal lymphocytic infiltration, cirrhosis, and bile duct proliferation.

In this laboratory cirrhosis was originally seen infrequently in hamsters by Dr. L. M. Franks and Mr. P. C. Williams in 1954. We have been aware of a high incidence of cirrhosis in both experimental and stock hamsters in recent years, and liver carcinomas are not infrequent (Chesterman, 1962 ; Chesterman and Pomerance, 1963, 1964*a*, *b*). In this communication the incidence and pathology of these conditions in our hamster colony are described, and the possible aetiological factors discussed.

### MATERIALS AND METHODS

The hamster colony has been developed from 6 male and 6 female golden hamsters obtained from the National Institute for Medical Research in 1953. The original hamsters at the National Institute were descendants of those presented to the Pathology Department of the University of Jerusalem by Professor Aharoni.

The main colony at our Institute is propogated as a closed but not deliberately inbred colony. The general husbandry has been described by Menzies (1961). From 1953 to 1961 the hamsters were fed on pellet diet 41B and from 1961 onwards on pellet diet GR25 or GR2 supplemented with Saval puppy dog biscuit. They were also given green stuff and water *ad libitum*. In addition to the main colony we have established two inbred lines, one golden strain ICRF/GH now in the 29th generation and one white strain ICRF/WH2 now in the 20th generation (Burdette, 1963). The latter originated from crossing a white hamster obtained from the National Institute for Medical Research with a golden hamster from our colony.

Cirrhosis and liver tumours occur in the inbred strains but as the total numbers in each of these groups of animals is small, the incidence in all the hamsters will be described in this communication.

The autopsy records and relevant histological sections of all hamsters born after January, 1958, were reviewed, ending with last autopsy performed in 1963. Most of the diagnoses of cirrhosis were made on the characteristic gross appearance of the liver and confirmed by histology. The diagnosis of early cirrhosis was however not reliable grossly, being difficult to distinguish from passive congestion of the liver, and diagnosis of these cases was only made from the histological appearances. Since the earlier autopsies were performed by several people, and the problem of cirrhosis was not then under consideration it is likely that some of these mild cases were overlooked, and the incidence or cirrhosis found is therefore a minimum figure. Data on age, sex, experimental procedure (if any), date of birth and diet were obtained for each animal and the incidence of cirrhosis assessed in relation to these factors. The incidence of gallstones and presence of worm granulomas in the liver were also compared in cirrhotic and non-cirrhotic animals.

The total number of animals examined was 6221, (3286 female and 2935 male) comprising 888 untreated or stock breeding animals and 5333 animals which had been the subject of a variety of experimental procedures, mainly related to transplantation of human tissues, human, hamster and other rodent tumours, work on tumour viruses, and the effects of hormones. Most of these (5604) were over 6 months of age at the time of autopsy.

### RESULTS

The incidence of cirrhosis appears to have risen from less than 1% in 1958 to over 20% in 1963 (Fig. 1). It is probable that the steep rise in the early years is exaggerated. At this time our previously small colony was increased and moved to new quarters and the increase must be partly attributed to the increased numbers of animals available for examination, and the greater age to which it was possible to keep them.

FIG. 1.—Incidence of cirrhosis in the hamster colony 1958 to 1963.

*Sex*

In both the stock and experimental animals, it was found that cirrhosis was commoner in females. Almost one third of the female hamsters born in 1962 and killed over the age of 6 months were found to have cirrhosis (Fig. 2).

FIG. 2.—Sex differences in the incidence of cirrhosis.

*Effect of experimental procedure*

The rising incidence of cirrhosis was as marked in the stock and breeding hamsters as in the animals used for experimental purposes. Neither the type of experimental procedure, nor the route of inoculation appeared related to cirrhosis (Fig. 3).

*Age*

The incidence of cirrhosis increases sharply with age in both sexes (Fig. 4), but the incidence in young animals is increasing. In hamsters born before 1962 cirrhosis was extremely rarely seen under the age of 6 months, and we have therefore only considered animals surviving longer than this in assessing the relation of all the other factors to cirrhosis. The animals under 6 months of age shown in Fig. 4 therefore only comprise those born in 1962 or 1963, at which time cirrhosis was becoming apparent in an appreciable number of hamsters in this age group.

*Pathology*

Grossly, most cases showed fairly uniform nodularity of all lobes of the liver, both on external and cut surfaces. The liver consistency was firmer than normal. In a few cases complete atrophy of one lobe was present, with marked nodularity of the remaining lobes. In others, the gross appearances were indistinguishable

FIG. 3.—Comparison of incidence of cirrhosis in stock hamsters and those used for experiments.

FIG. 4.—Age and sex incidence of cirrhosis.

TABLE I.—*Type Number and Distribution of Liver Tumours According to Sex and Year of Birth in Hamsters over* 6 *Months Old* 1958–1963

| Year of birth | Number of Autopsies on animals aged over 6-months | | | Cholangiocarcinoma | | Hepatoma | | Other tumours | |
|---|---|---|---|---|---|---|---|---|---|
| | Male | Female | Total | Male | Female | Male | Female | Male | Female |
| 1958 | 655 | 908 | 1563 | 0 | 0 | 0 | 1 | 0 | 0 |
| 1959 | 608 | 580 | 1188 | 0 | 2 | 0 | 0 | 0 | 0 |
| 1960 | 544 | 530 | 1074 | 3 | 5 | 0 | 0 | 0 | 1 |
| 1961 | 561 | 553 | 1114 | 3 | 9 | 2 | 0 | 1 | 1 |
| 1962 | 220 | 312 | 532 | 2 | 4 | 1 | 2 | 0 | 0 |
| 1963 | 56 | 77 | 133 | 1 | 4 | 0 | 0 | 1 | 0 |
| | | | | 9 | 24 | 3 | 3 | 2 | 2 |
| Totals | 2644 | 2960 | 5604 | 33 | | 6 | | 4 | |

from passive congestion of the liver, but the majority of livers with cirrhosis showed the appearances seen in the non tumour-bearing area of Fig. 5. Oedema, pleural effusions, and ascites were sometimes observed in animals with severe cirrhosis. Pale granular kidneys were observed particularly in old animals. Plasma proteins from a few of the hamsters with cirrhosis were examined and showed reversal of the albumin globulin ratio and an increase in the 17S macroglobulin. The triad of liver lesions, renal disease and hypergammaglobulinaemia is similar to some of the pathological findings in Aleutian disease of mink (Leader and Wash, 1964). Microscopically most cases showed a well developed fine multilobular cirrhosis, in which bile duct proliferation was the most prominent feature. Broad bands of tightly packed regular acini separated small groups of liver lobules (Fig. 6). The quantity of fibrous tissue varied, but was exceeded by the bile duct proliferation. Almost acellular hyalinised bands of connective tissue separating groups of lobules were less frequently seen. Some stained matachromatically for amyloid. This type of cirrhosis was confined to the very old hamsters. In the early stages there was extensive periportal cellular infiltration, mainly by lymphocytes, with moderate numbers of plasma cells and sometimes eosinophils. Hepatocellular degeneration was not prominent in most of the sections examined, although in an occasional animal degeneration and nodular regeneration with cellular pleomorphism and binucleate cells were a conspicuous feature.

Although most of the bile duct proliferation was of the regular histologically benign pattern, shown in Fig. 6, occasionally small areas of atypical proliferation were seen. Irregularly-shaped acini were found in fibrous stroma, and the cells of these acini were pleomorphic (Fig. 7) frequently with hyperchromatic nuclei, and occasional mitoses. Morphologically some of these areas were indistinguishable from the cholangiocarcinomas which were apparent macroscopically (Fig. 5).

EXPLANATION OF PLATES

FIG. 5.—Autopsy appearance of liver with cirrhosis and a cholangiocarcinoma. ×1·5.

FIG. 6.—Typical appearance of cirrhotic liver with bile duct proliferation. H. & E. ×120.

FIG. 7.—Atypical bile duct proliferation in a cirrhotic liver. H. & E. ×110.

FIG. 8.—Metastatic cholangiocarcinomas in hamster lung in an animal bearing a subcutaneous transplanted cholangiocarcinoma. H. & E. ×110.

FIG. 9.—Hepatoma showing parenchymal liver cells irregular in size and shape. H. & E. ×110.

FIG. 10.—Periportal cellular infiltration, fibrosis and bile duct proliferation in a hamster from another colony. H. & E. ×110.

5

6

7

8

Chesterman and Pomerance.

9

10

Chesterman and Pomerance.

### *Tumours in Cirrhotic Livers*

### *Incidence*

Forty-seven primary tumours of the liver have been noted in our colony in the period under review. All except 3 of these were in animals over 6 months of age. The types, numbers and distribution according to sex and year of birth of these are shown in Table I. Thirty-three were classified as cholangiocarcinomas, 6 as hepatomas, and 1 showing both types of tumour. In addition there were 4 animals in which the liver tumour sections had the histological appearance of a metastatic tumour but no primary had been identified elsewhere.

As would be anticipated from the incidence of cirrhosis, the increasing incidence of tumours was seen and paralleled the increase in cirrhosis. Also as with cirrhosis, females were more commonly affected, the numbers in both sexes increased with increasing age. Tumours, like cirrhosis, appeared in young animals born in recent years. The 3 animals found to have cholangiocarcinomas under the age of 6 months were all born in 1963.

### *Pathology*

Marked bile duct proliferation was the most striking feature of the cirrhosis and most of the tumours seen were cholangiocarcinomas. Grossly, they appeared as firm grey nodules, mainly subcapsular in distribution, and varying from pinhead to 1½ cm. diameter (Fig. 5), and were frequently multiple. Microscopically these areas showed irregular acini of large flat pleomorphic epithelial cells with hyperchromatic nuclei, and occasional mitoses, in a dense fibrous stroma. Subcutaneous transplantation of 6 of these tumours was successful in 4 cases although one became infected and was discontinued after the 4th transfer. Another was discontinued at the 32nd transfer. The remaining two were stored in the frozen state after the 5th and 28th transfer respectively. In one of these lines progressive increase in malignancy developed, and by the 11th transfer this tumour appeared as very poorly differentiated highly cellular columnar celled adenocarcinoma with numerous mitoses. Metastases to regional lymph nodes and lungs were also seen in some of the animals bearing transplanted tumours (Fig. 8).

Owing to the difficulty in differentiating between hepatomas and nodular regeneration, a diagnosis of hepatoma was only accepted where the degree of disorganisation of architecture, cellular pleomorphism and numbers of binucleate cells and mitotic figures were such that the neoplastic nature of the nodule was unequivocal (Fig. 9). These criteria were fulfilled by 6 tumours. Grossly the hepatomas appeared as solid, soft, dark red spherical masses, poorly demarcated from the surrounding liver tissue. Transplantation has not been attempted in these cases.

In addition to tumours which could be easily classified as cholangiocarcinomas or hepatomas, there were 4 cases in which the histology showed well-differentiated papillary columnar celled pattern. In 2 of these the appearances were unlike any of the usual primary carcinomas of liver and strongly suggestive of a metastatic tumour, but no other primary source had been found at autopsy. In the remaining 2, one resembled the cholangiocarcinoma after repeated transplantation and the other was a mucus-secreting tumour similar to some of the small islands of atypical bile duct proliferation seen in many of the cirrhotic livers ; these two cases were therefore considered to be primary liver tumours of bile duct origin.

### DISCUSSION

The rather steep rise in the incidence of cirrhosis seen in the first years, as shown in the graph (Fig. 1), is partly due to factors other than a true increase in cirrhosis.

Although cirrhosis was seen in the older animals at and before 1958, there was no particular interest in this disease, and since it was unrelated to work then in progress it is likely that no record of cirrhosis was made at autopsy in a number of animals in which this finding had actually been observed. However, those factors did not apply after the second of the 6 years under review. The estimated incidence of cirrhosis then was probably still too low, since early cases may show no macroscopic abnormality but the degree of underestimation is likely to be constant over the past 5 years, and therefore any changes in incidence shown by the graph reflect the increasing frequency with which cirrhosis is occurring in our hamster colony.

The aetiology of this disease is not clear at present. The proportion of animals affected is increasing, and cirrhosis is also appearing in increasing numbers of young animals, and since our colony is a closed one, all deriving from an original nucleus of 12 animals, the possibility of a genetic determination must be considered. It may be that we are breeding a strain of hamster which is becoming increasingly liable to spontaneous, idiopathic, cirrhosis, or that the animals are developing an increasing susceptibility to some hepatic insult, which we have not yet been able to identify. The histological features are similar to those described by Farber (1956) in rats receiving hepatic carcinogens.

In connection with a possible viral aetiology it is interesting to observe that Hlavay and Svec (1965) found hepatitis followed by cirrhosis in a large number of inoculated rats used in attempts to transplant chronic myelogenous chloroleukaemia by cell free inoculation.

With regard to the increased incidence of cirrhosis in females Dontenwill and Mohr (1961) found that cystic bile duct proliferation induced by acetoaminofluorene in hamsters was inhibited by testosterone and potentiated by F.S.H. Ising (1956) found more thin-walled cysts in the livers of stilboestrol treated males than in similarly treated female hamsters. We have seen similar cysts in stock and experimental animals of both sexes.

Male and female hamsters also respond differently to an oncogenic virus. Yohn, Funk and Grace (1965) have found the incidence of tumours induced by adenovirus type 12 is higher in females than males.

Apart from the published reports previously mentioned, we have made enquiries as to the incidence of cirrhosis in hamsters of other colonies but have been unable to find much information on animals over 6 months. A similar kind of cirrhosis (Fig. 10) to that seen in our hamsters occurred in a few hamsters at University College Hospital Medical School. These were obtained by Dr. Butler from a dealer and were fed on a diet containing less than 0·04 parts per million aflatoxin. These animals were used as a control group in experiments on the effects of aflatoxin in hamsters. Similar lesions were seen in the treated animals receiving 2·0 parts per million aflatoxin.

Cirrhosis has also been observed in one colony of hamsters in the U.S.A. (Russfield, 1965, personal communication).

We have also considered, and failed to find, any relation between enteritis, or gall stones, and cirrhosis. Attempts were made to relate cirrhosis to definite epidemics of enteritis, or to animals in groups of cages in which enteritis had occurred, but no relationship was established. It must, however, be admitted that outbreaks of clinical enteritis are common in our colony, and subclinical infections may in fact have occurred in almost all the animals. We have as yet no information on the incidence of cirrhosis in hamsters living under pathogen-free conditions.

Gall stones are easily identifiable at autopsy, and the possibility that the cirrhosis was due to cholangitis was explored. Although dilated intrahepatic bile ducts containing masses of polymorphs were not infrequently seen in sections of cirrhotic livers, the incidence of gall stones in the colony was very small in relation to the incidence of cirrhosis, and was not increasing in frequency. The stones were, if anything, more frequent in hamsters with otherwise normal livers than in those with cirrhosis. Microscopic examination of the gall-bladders also failed to show any significant incidence of cholecystitis.

Proliferation of bile duct epithelium occurs in rabbits infected with *Eimeria stiedae* (see Gresham and Jennings, 1962) and tumours of the liver are known to occur in association with *Cysticercus fasciolaris* in rats (Bullock and Curtis, 1920), *Bilharzia mansoni* infections in mastomys (Oettlé, de Meillon and Lazer, 1959), and *Chlonorchis sinensis* infestation in cats and man (Hou, 1964 ; Hou and Pang, 1964). It is possible that the cirrhosis in our colony may be parastic in origin. Fragments of the larval stage *Cysticercus fasciolaris* have been seen on a few occasions. Infestation with a cestode of the genus *Hymenolepis* is endemic in our hamsters. These parasites have been seen in the duodenum, small intestine, pancreatic, extra- and intra-hepatic bile ducts, and mesenteric nodes. Worm granulomas are not uncommonly seen in routine liver sections. Experimental infections of hamsters with *Hymenolepis microstoma* produces enteritis and fibrotic lesions of the liver (Dvorak, Jones and Kuhlman, 1961 ; Litchford, 1963). However, there does not at present appear to be any quantitative relationship between the incidence of worm granuloma and cirrhosis, the granulomas being more frequent in non-cirrhotic livers, since a causative parasite is unlikely to be visible once cirrhosis has developed. Absence of worm fragments or granulomas is of doubtful significance and a study of animals known to be free from internal parasites will be necessary before the role of helminth infestations in cirrhosis can be assessed.

Another possibility is that some factor in the diet is responsible for the cirrhosis. In the course of experiments on the effects of diets including toxic groundnut meal, it was noticed that the control animals fed on the normal laboratory pellet diets had a higher incidence of cirrhosis than those fed on either the toxic or nontoxic diets obtained from an outside source. The total number of hamsters concerned is not high enough for a definite conclusion to be drawn, but of the 45 hamsters fed on our stock diets, 31 developed cirrhosis (68%) and 32 out of 60 fed on an imported diet (53%). If a dietary factor is responsible, it is probably not aflatoxin since batches of our diet have been tested and not found to contain sufficient aflatoxin to affect the standard test ducklings. However, other moulds found in grains have been shown to have hepatotoxic and oncogenic action in laboratory animals (Kraybill and Shimkin, 1964), and it is possible that these or similar substances are present in our diets. It is interesting to note that the change in incidence of cirrhosis corresponds to a change in diet formula in 1961. The formula has been revised again recently, and groundnut meal excluded because of the possibility of contamination with the aflatoxin producing mould *Aspergillus flavus*. Hamsters which have been fed only on this new formula are as yet too young for the incidence of cirrhosis to be assessed, but it is evident that the presence or absence of any toxic factor in the groundnut meal should be easily determinable once these animals reach an age where a high incidence of cirrhosis was previously seen (i.e. 1–2 years), and we shall be reporting our findings on these animals in due course.

SUMMARY

The pathology of cirrhosis and tumours of the liver observed during a period of five years in a closed colony of golden hamsters is described.

888 untreated hamsters and 5333 hamsters used in a variety of experimental procedures were examined. The incidence of cirrhosis appeared to increase from less than 1% in 1958 to over 20% in 1963. It was commoner in females (33%) compared with 20% in males), increased with age, and was uninfluenced by experimental procedures. Microscopy showed a multilobular portal cirrhosis with bile duct proliferation as the predominant feature. 6% of cirrhotic livers had developed tumours ; and in animals over 6 months of age 33 were cholangiocarcinomas, 6 were hepatomas, 1 contained both types of tumour and in 4 the origin of the tumour could not be determined. Attempts to transplant some of the cholangiocarcinomas were successful.

The aetiology of the cirrhosis was not clear. It appeared unrelated to epidemics of enteritis and to gall stones. Helminth infestation is endemic in the colony, and the possibility that cirrhosis is a result of this cannot be excluded. Dietary factors are suggested by the finding that cirrhosis was less frequent in animals fed on an imported diet. Finally, since cirrhosis is appearing with increasing frequency in younger animals, it is possible that this condition is influenced by genetic factors.

We are grateful to Mrs. M. O. Phillips and staff for the sections, Messrs. E. V. Willmott and J. Pringle for the photographs. Dr. W. H. Butler for histological material, Dr. J. S. Paterson for the toxic groundnut meal. Mrs. Ruth Allcroft for testing our diet, Miss Frances Crew for examining the plasma proteins and Professor J. J.C. Buckley and Mr. F. R. N. Pester for identifying the worms. Also to Elizabeth von Laur for translation from the German literature.

### REFERENCES

BAIN, G. O., ALLEN, P. B. R., SILBERMANN, O. AND KOWALEWSKI, K.—(1959) *Cancer Res.*, **19**, 93.

BULLOCK, F. D. AND CURTIS, M. R.—(1920) *Proc. N.Y. path. Soc.*, **20**, 149.

BURDETTE, W. J.—(1963) In ' Methodology in mammalian genetics ', edited by Burdette. San Francisco (Holden-Day Inc.), p. 115.

CHESTERMAN, F. C.—(1962) *Int. Cancer Res. Congr. Abst. of Papers*, Moscow (Medgiz) p. 486.

CHESTERMAN, F. C. AND POMERANCE, A.—(1963) *Rep. imp. Cancer Res. Fund*, **61**, 59.—(1964a) *Ibid.*, **62**, 46.—(1964*b*) *Ned. Tijdschr. Geneesk.*, **108**, 2380.

CRABB, E. D. AND KELSALL, M. A.—(1951) *Cancer Res.*, **11**, 243.

DONTENWILL, W. AND MOHR, V.—(1961) *Z. Krebsforsch.*, **64**, 388.

DVORAK, J. A., JONES, A. W. AND KUHLMAN, H. H.—(1961) *J. Parasit.*, **47**, 833.

FARBER, E.—(1956) *Cancer Res.*, **16**, 142.

FORTNER, J.—(1957) *Cancer, N.Y.*, **10**, 1153.
FRENKEL, J. K. AND HAVENHILL, M. A.—(1963) *Lab. Invest.*, **12**, 1204.
GALTON, M.—(1964) *Am. J. Path.*, **44**, 613.
GRESHAM, G. A. AND JENNINGS, A. R.—(1962) 'An Introduction to Comparative Pathology', London & New York (Academic Press), p. 138.
HLAVAY, E. AND SVEC, F.—(1965) *Br. J. exp. Path.*, **46**, 164.
HOU, P. C.—(1964) *J. Path. Bact.*, **87**, 239.
HOU, P. C. AND PANG, L. S. C.—(1964) *Ibid.*, **87**, 245.
ISING, U.—(1956) *Acta path. microbiol. scand.*, **39**, 168.
KIRKMAN, H.—(1952) *Cancer Res.*, **12**, 274.
KLEMENT, V. AND SVOBODA, J.—(1963) *Folia biol. Kraków*, **9**, 181.
KRAYBILL, H. F. AND SHIMKIN, M. B.—(1964) *Adv. Cancer Res.*, **8**, 191.
LEADER, R. W. AND WASH, P.—(1964) *Archs Path.*, **78**, 390.
LITCHFORD, R. G.—(1963) *J. Parasit.*, **49**, 403.
MAGEE, P. N.—(1964) *J. natn. Cancer Inst.*, **33**, 341.
MALIUGINA, L. L.—(1958) *Probl. Oncol.*, **4**, 296.
MENZIES, J. D. E.—(1961) *J. Anim. Techns Ass.*, **12**, 1.
OETTLE, A. G., DE MEILLON, B. AND LAZER, B.—(1959) *Acta Un. int. Cancr.*, **15**, 200.
PASCHKIS, K. E., CANTAROW, A. AND GODDARD, J. W.—(1957) *Proc. Am. Ass. Cancer Res.*, **2**, 238.
PORTA, D. G., SHUBIK, P. AND SCORTECCI, V.—(1959) *J. natn Cancer Inst.*, **22**, 463.
PORTA, D. G., TERRACINI, B. AND SHUBIK, P. (1961) *Ibid.*, **26**, 855.
TERRACINI, B. AND PORTA, D. G.—(1961) *Archs Path.*, **71**, 566.
TOMATIS, L., MAGEE, P. N. AND SHUBIK, P.—(1964) *J. natn. Cancer Inst.*, **33**, 341.
YOHN, D. S., FUNK, C. A. AND GRACE, J. T. JR.—(1965) *Proc. Am. Ass. Cancer Res.*, **6**, 70.